# Feed Composition and Isolate of *Histomonas meleagridis* Alter Horizontal Transmission of Histomonosis in Turkeys. Proof of Concept

**DOI:** 10.3389/fvets.2022.937102

**Published:** 2022-06-28

**Authors:** Thaina L. Barros, Christine N. Vuong, Juan D. Latorre, Roberto S. Cuesta, Elizabeth McGill, Samuel J. Rochell, Guillermo Tellez-Isaias, Billy M. Hargis

**Affiliations:** ^1^Center of Excellence for Poultry Science, University of Arkansas, Fayetteville, AR, United States; ^2^Cargill Turkeys LLC, Springdale, AR, United States

**Keywords:** intestinal health, enterohepatitis, protozoa, ceca, epidemiology

## Abstract

Outbreaks of histomonosis in turkeys are typically initiated by the ingestion of contaminated embryonated eggs of *Heterakis gallinarum*, potentially present in earthworms and mechanical vectors. Once an outbreak is started, infected turkeys can transmit the disease by horizontal transmission. Factors influencing horizontal transmission of histomonosis are poorly understood. Replication of horizontal transmission in experimental conditions has not been consistent, presenting an obstacle in searching for alternatives to prevent or treat the disease. Two pilot experiments and three validation experiments were conducted in the present study. In pilot experiment 1, one isolate of *Histomonas meleagridis* (named Buford) was used. Turkeys were fed a low-nutrient density diet corn-soy based (LOW-CS) and raised in floor pens. In pilot experiment 2, another isolate of *H. meleagridis* was used (named PHL). Turkeys were fed a low-nutrient density diet with the addition of wheat middlings (LOW-WM) and raised in floor pens. In experiment 3, conducted on floor pens, both isolates and diets were used in different groups. In experiment 4, turkeys were raised on battery cages and only the PHL isolate was used. Both diets (LOW-WM and LOW-CS) were used, in addition to a diet surpassing the nutritional needs of young poults (turkey starter, TS). In experiment 5, conducted in battery cages, only the PHL isolate was used, and the LOW-WM and TS diets were in different groups. The horizontal transmission was achieved only with the PHL isolate from all experiments. The transmission rate varied among experimental diets, with the TS diet having the lowest transmission rate in experiments 4 and 5. Variation was observed between experiments and within experimental groups.

## Introduction

The incidence of histomonosis, caused by the protozoa *Histomonas meleagridis*, has been increasing after the ban of nitroimidazoles and arsenicals as preventative and therapeutic measures (1). Outbreaks can lead to high mortality rates in turkey flocks (2–4). Turkeys become infected by ingesting contaminated *Heterakis gallinarum* eggs, but once a turkey is infected, the transmission to other turkeys can happen in the absence of *H. gallinarum* by direct contact (5), referred to in the present paper as horizontal transmission.

In the last decade, there has been an increase in research about histomonosis (2, 3, 6); however, some epidemiologic aspects remain unanswered, such as risk factors associated with the transmission of histomonosis and the lack of a model of infection by horizontal transmission. The replication of horizontal transmission of histomonosis has not been consistent in many research groups in the last years (6). One explanation is the duration of the trials, with experiments ending before turkeys could show lesions (6). In outbreaks of histomonosis in turkey flocks, it is common to have some variability, whereas, in some outbreaks, only females or only males were affected in a mixed flock house, typically separated by a wire mesh. Factors behind the variability in disease manifestation and incidence are unknown. Previous studies have reported the existence of two distinct clusters of *H. meleagridis* differing in prevalence in geographical locations and pathology (7) and variation in the expression of virulence factors and pathogenicity of different field isolates (8). Nevertheless, as far as we are aware, the impact of different isolates of *H. meleagridis* or the feed composition of the diet in the horizontal transmission of the disease has not been investigated. Hence, the purpose of this study was to evaluate the feed composition and isolates of *H. meleagridis* on the transmissibility of histomonosis in floor pens or battery cages.

## Materials and Methods

### Bioethics

All animal handling procedures complied with the University of Arkansas Agricultural Institutional Animal Care and Use Committee (Animal Use Protocol #19118). Following inoculation with *H. meleagridis*, turkeys were monitored at least twice daily to evaluate and potentially euthanize terminally moribund animals. Severe clinical morbidity, with evidence of inability to ambulate, were euthanized and considered as mortalities.

### Pathogen Culture and Challenge

The Buford isolate consists of a wild-type *H. meleagridis* from a field outbreak of histomonosis in layer pullets in the southern United States (9). The PHL isolate is also a wild-type *H. meleagridis*, isolated in 2017 from turkeys in the Northwest Arkansas, USA. Both isolates were cultured in Medium 199 (Lonza^®^, Walkersville, Maryland) supplemented with 10% horse serum (Gibco^®^, Penrose, Auckland, New Zealand) and 1.6 mg/mL rice powder (Arrowhead Mills^®^, Hereford, Texas), following previously described procedures (10). The 1mL vial cryogenically stored from each isolate was thawed and cultured in T-25 cell culture flasks (12.5mL culture volumes) approximately 1 week (Buford isolate) and 2 weeks (PHL isolate) before the inoculation. Cultures were incubated anaerobically at 40°C and passaged every 2 to 3 days. On the inoculation day for each experiment, histomonads were counted with a hemocytometer, adjusting the inoculum to 10^5^ histomonads/dose using unsupplemented media. The volume of the inoculum varied between 250–500 μL between the trials, always administered via the intra-cloacal route, holding turkeys in an inverted position for 1 min after the inoculation to reduce the possibility that the turkeys would expel the material.

### Animal Source and Husbandry

In all experiments, turkeys had *ad libitum* access to feed and water. All turkeys were female and were obtained from a commercial hatchery (Cargill, Gentry, AR, USA) for experiments 1, 2, and 3 and from another commercial hatchery (Butterball, Goldsboro, NC, USA) for experiments 4 and 5.

### Experimental Design

#### Pilot Studies

In the present study, two pilot experiments were conducted with the intention of assessing the horizontal transmission of two different isolates of *H. meleagridis* and two diet compositions in turkeys.

In the first pilot study (Experiment 1), the inoculation of a typical isolate of *H. meleagridis* (Buford) and the use of a low-nutrient density diet with a reduction in the crude protein level would cause transmission of histomonosis in turkeys raised on floor pens. Pilot experiment 1 was conducted on floor pens (4.5 m^2^), with wood shavings as the bedding material. Fifty day-of-hatch poults were randomly distributed to either a non-challenged control (NC) or horizontal transmission group (HT), both being fed a mashed low-nutrient density (LOW) diet, corn-soy based (CS) diets divided into two phases: the first CS diet (CS1) from day 10 to day 21 and the second CS diet (CS2) from day 21 to 45. The diets were formulated based on requirements for broilers (broiler starter and developer), with a lower protein content compared to a turkey diet commonly used in trials with turkeys conducted by our group, which surpasses the nutritional needs of young turkeys, referred to as turkey starter (TS). The composition of the diets is available in Table 1. The turkey starter diet was fed during the first 10 days of the poults' life. Seven out of twenty-five turkeys were directly inoculated on day 14 with the Buford isolate (10^5^ histomonads/turkey), referred to as seeders. The experiment was terminated on day 45; mortality was monitored daily, and hepatic and cecal lesions were evaluated on a scale of 0–3 (10). The following experiments followed the same criteria of evaluation.

**Table 1:** Composition of the experimental diets.

**Ingredient (%)**	**Low-nutrient density diet corn soy based (LOW-CS)**		**Diet surpassing the nutritional needs of young turkeys (TS)**	**Low-nutrient density diet with wheat middlings (LOW-WM)**
	**Corn-soy 1 (CS1)**	**Corn-soy 2 (CS2)**		
Corn	57.90	75.64	43.33	61.75
Soybean meal	30.23	19.09	42.24	13.20
Wheat middlings	-	-	-	20.50
Animal protein concentrate^§^	5.00	0	7.50	-
Poultry fat	3.58	1.00	3.40	-
Limestone	1.10	1.59	0.66	-
Calcium	-	-	-	1.52
Monocalcium phosphate	-	-	-	2.13
Dicalcium phosphate	1.10	1.57	1.52	-
Salt	0.40	0.41	0.24	0.25
Bicarbonate	-	-	-	0.20
Methionine	0.20^1^	0.20^1^	0.38^1^	0.16^3^
Lysine	-	-	0.42^2^	0.02^4^
L-threonine	-	-	0.11	-
Vitamin/mineral premix	0.20/0.10^†^	0.20/0.10^†^	0.15^†^	0.23^e^
Choline chloride (60%)	0.20	0.20	0.05	0.02^£^
Enzymes^¶^	-	-	-	0.02
**Calculated composition (%)**				
Crude protein	22	15	28	14
AME (kcal/kg)	3,098	3,082	3,020	2,800
Total Ca	1.27	1.07	1.49	1.15
Available phosphorus	0.56	0.43	0.76	0.58
Dig TSAA	0.82	0.67	1.06	0.55
Dig Lys	1.02	0.69	1.64	0.62
Dig Thr	0.68	0.49	0.96	0.43
Dig Ile	0.79	0.56	1.01	0.49
Dig Val	0.91	0.66	1.12	0.59
Dig Trp	0.22	0.15	0.28	0.14
Dig Arg	1.34	0.88	1.75	0.80

^§^*Composition: crude protein, 57%; crude fat, 8.5%; calcium, 7.94%; phosphorus, 3.59%; sodium, 0.49%; potassium, 0.38%; chloride, 0.73%; cysteine, 1%; methionine, 0.71%; lysine, 3.13%; histidine, 0.91%; tryptophan, 0.34%; threonine, 1.97%; arginine, 3.78%; isoleucine, 1.88%; leucine, 3.71%; phenylalanine, 2.09%; valine, 2.77% (H.J. Baker's ProPlus 57%)*.

^†^*Supplied per kg of feed by vitamin premix (0.2%): Vitamin A, 61,740 IU; vitamin D3, 44,100 ICU; vitamin E, 441 IU; vitamin B12, 0.1 mg; menadione, 12 mg; riboflavin, 52.9 mg; D-pantothenic acid, 79.4 mg; niacin, 308.6 mg; folic acid, 7.1 mg; pyridoxine, 22 mg; thiamine, 12.3 mg; biotin, 0.7 mg. Mineral premix (0.1%): calcium, 767 mg; total phosphorus, 0.8 mg; potassium, 1.2 mg; sodium, 1.2 mg; magnesium, 1.0 mg; sulfur, 1,228 mg; iron, 15 mg; zinc, 100 mg; manganese, 100 mg; copper, 15 mg; iodine, 1.2 mg; selenium, 0.3 mg*.

^‡^*Supplied per kg of feed by vitamin and mineral premix (0.15%): Vitamin A, 13,227 IU; vitamin D3, 6,613 ICU; vitamin E, 66 IU; calcium, 51 mg; manganese, 124.5 mg; zinc, 124.5 mg; copper, 7.5 mg; iodine, 2.1 mg; selenium, 0.3 mg*.

^e^*Supplied per kg of feed by Vitamin and mineral premix (0.225%) Vitamin A, 13,230 IU; vitamin D3, 66,100 ICU; vitamin E, 100 IU; vitamin B12, 0.0248 mg; vitamin E EQ, 100 mg; biotin, 0.33 mg; menidione, 4 mg; riboflavin, 15 mg; d-pantothenic acid, 24.26 mg; niacin, 88.2 mg; folic acid, 1.1 mg; pyridoxine, 6.9 mg; thiamine, 2.21 mg; manganese, 125.1 mg; chelated manganese, 40 mg; zinc, 125.1 mg; chelated zinc, 40 mg; iron, 2.1 mg; copper, 7.5 mg; iodine, 2.1 mg; selenium, 0.3 mg*.

^£^*Choline chloride (70%)*.

^¶^*Rovabio^®^ Advance Phy L: endo-1,4-β-xylanase ≥ 6,250 VU/ml, endo-1,3(4)-β-glucanase ≥ 4,300 VU/ml, arabinofuranosidase ≥ 23,000 VU/ml, 6-phytase ≥5,000 FTU/ml*.

^1^*DL-methionine*.

^2^*L-lysine HCl*.

^3^*Methionine hydroxy analog (88% methionine)*.

^4^*BIOLYS-77^®^ (60% lysine)*.

In the second pilot study (Experiment 2), we used a different isolate of *H. meleagridis*, named PHL, and a low-nutrient density diet with the addition of 20.5% wheat middlings provided by a commercial company that frequently reports outbreaks of histomonosis in pre-reproductive turkey hens. Sixty-eight day-of-hatch poults were randomly distributed to either a non-challenged control group (*n* = 34) or a horizontal transmission group (*n* = 34). Ten of the 34 poults in the HT group were directly inoculated (seeders) on day 18 with only the PHL isolate (10^5^ histomonads/turkey). Poults were fed the mashed turkey starter diet for the first 14 days, and on day 15, a pelleted LOW diet containing 20.5% wheat-middlings (WM) was introduced until the end of the experiment (day 52). The experiment was conducted on floor pens. The composition of the diet is available in Table 1. The experiment was terminated on day 52; mortality was monitored daily, with an evaluation of the lesions.

#### Validation Studies

Based on the findings of the pilot studies, three experiments were conducted to validate the preliminary data as described below.

In Experiment 3, also conducted in floor pens, two LOW diets (CS and WM) with the same formulation used in the first two experiments were introduced after the first week of the poults' lives: WM diet (WM, d7-38) and CS diets (CS1, d7-21, and CS2, d21-38). The diets were similar to those in the first two experiments, but in different batches. All poults were fed the turkey starter diet during the first seven days. The two previously mentioned isolates of *H. meleagridis* were tested: Buford or PHL. Day-of-hatch poults were randomly distributed to one of eight groups: 1) NC, fed a LOW diet, CS-based (NC-CS, *n* = 45); 2) NC, LOW diet, WM based (NC-WM, *n* = 50); 3) positive control (PC), all turkeys directly inoculated with the Buford isolate, fed a LOW diet, WM based (PC-Buford-WM, *n* = 50); 4) PC, all turkeys directly inoculated with the PHL isolate, fed a LOW diet, WM based (PC-PHL-WM, *n* = 50); 5) HT with the Buford isolate, fed a LOW diet, WM based (HT-Buford-WM, *n* = 45); 6) HT with the Buford isolate, fed a LOW diet, CS-based (HT-Buford-CS, *n* = 45); 7) HT with the PHL isolate, fed a LOW diet, WM based (HT-PHL-WM, *n* = 45); or 8) HT with the PHL isolate, fed a LOW diet, CS-based (HT-PHL-CS, *n* = 45). Due to a space limitation, we did not include positive controls with the CS diet. On day 10, 14 of 45 poults in the HT groups were directly inoculated with 10^5^ histomonads/turkey and all turkeys (*n* = 50/group) in the PC groups. Mortality was recorded daily in both experiments. The PC-Buford-WM was terminated on day 24, 14 days post infection (d.p.i.), the PC-PHL-WM on day 36 (26 d.p.i.), and the remaining groups on day 38; mortality was monitored daily, and hepatic and cecal lesions were evaluated on a scale of 0–3. Bodyweight gain (BWG) from day 7 to 38 was measured only on the NC groups because the PC-Buford-WM group had to be terminated on day 24, and the PC-PHL-WM was terminated on day 36. For those groups, BWG was measured from day 7 to day 21.

In Experiment 4, turkeys were raised in battery cages. The cage floor was covered with heavy paper from day 10 to day 25. The paper was changed daily. Three diets were tested: in addition to the two previously LOW diets (WM and CS), the diet surpassing the nutritional needs of young turkeys (turkey starter, TS) was used throughout the experiment for two groups. The TS diet was administered to all groups for the first seven days, then the WM diet was introduced on day 7 until termination (d30) (groups 2 and 5), or the CS diet was divided into two phases (CS1 and CS2), or the TS the whole period (groups 1 and 4). The CS1 was administered from day 7 to day 21 and CS2 from day 21 to termination (d30) (groups 3 and 6). One-hundred ninety-two poults were randomly divided into the following groups (*n* = 8 poults/cage, 4 replicates): 1) NC, fed the TS diet (NC-TS); 2) NC, fed a LOW diet, WM based (NC-WM); 3) NC, fed a LOW diet, CS-based (NC-CS); 4) HT group, fed the TS diet (HT-TS); 5) HT group, fed a LOW diet, WM based (HT-WM); or 6) HT group, fed a LOW diet, CS-based (HT-CS). Only the PHL isolate was used; on day 9, 2 of 8 poults were directly inoculated (seeders) with 10^5^ histomonads/turkey. Body weight was recorded on day 7 and at termination (day 30) to calculate BWG. Mortality was recorded daily with an evaluation of lesions.

In Experiment 5, turkeys were again raised in battery cages, following the abovementioned practices. For this trial, only two diets were used: the LOW diet, WM-based with the addition of 3% of celite as a filler, and the corn-soy diet surpassing the nutritional needs of young turkeys (TS), mashed. The TS diet was administered to all groups for the first seven days, then the WM diet was introduced on day 7 until termination (d29) (groups 2 and 4) or the TS diet for the whole period (groups 1 and 3). Two hundred forty poults were randomly allocated into 4 groups (*n* = 10/cage, 6 replicates): 1) NC, fed the TS diet (NC-TS); 2) NC, fed a LOW diet, WM based (NC-WM); 3) HT group, fed the TS diet (HT-TS); or 4) HT group, fed a LOW diet, WM based (HT-WM). On day 7, only 8 poults were kept in each cage. On day 9, 2 of 8 poults were directly inoculated (seeders) with 10^5^ histomonads/turkey from the PHL isolate. Body weight was recorded on day 7 and at termination (day 27) to calculate BWG, and feed consumption was recorded. Mortality was recorded daily with an evaluation of lesions.

### Statistical Analysis

Mortality and frequency of lesions were compared with all possible combinations using the chi-square test of independence to determine significance in experiment 3, 4 and 5 (*P* < 0.05). Bodyweight gain data were subjected to multi-way analysis of variance for the randomized design using SAS's General Linear Models procedure. Means were separated with the Duncan test and considered significant at *P* < 0.05. Data were reported as mean ± SE. In experiment 3, for BWG each bird was the experimental unit. In experiments 4 and 5, the average of each cage was considered the experimental unit for performance data and individual birds for lesions scores. For all experiments, turkeys succumbing to infection or which were euthanized were subjected to lesion scoring.

## Results

Table 2 shows a summary of the results of the five horizontal transmission experiments in turkeys fed with different feed compositions and challenged with two different isolates of *Histomonas meleagridis*.

**Table 2:** Summary of the results of the horizontal transmission, evaluated as the % frequency of lesions in ceca and/or liver in contact turkeys for all five experiments evaluating different feed compositions and isolates of *Histomonas meleagridis*.

**Experiment**	**Diet**	**Isolate**	**Number of turkey seeders/Number of turkey contacts**	**% Horizontal transmission in contacts**	**% Mortality rate (seeders / total)**	**% Mortality rate** **(contacts / total)**
**Pilot experiment 1** **(Floor pen)**	CS^1^	Buford	7/18	0 %	100 % (7/7)	0 % (0/18)
**Pilot experiment 2** **(Floor pen)**	WM^2^	PHL	10/23	30.4 % (7/33)	40 % (4/10)	4.3 % (1/23)
**Experiment 3** **(Floor pen)**	WM CS	Buford or PHL	14/30	57 % (17/30) PHL-CS^a^ 0 % (0/30) PHL-WM^b^ 0 % (0/30) Buford-WM^b^ 0 % (0/30) Buford-CS^b^	42.9 % (6/14) PHL-CS 42.9 % (6/14) PHL-WM 64.3 % (9/14) Buford-WM 57.1 % (8/14) Buford-CS	23.3 % (7/30) PHL-CS^a^ 0 % (0/30) PHL-WM^b^ 0 % (0/30) Buford-WM^b^ 0 % (0/30) Buford-CS^b^
**Experiment 4** **(Battery cages)**	WM CS TS^3^	PHL	2/6	100 % (23/23) WM^a^ 83.3 % (20/24) CS^b^ 45.8 % (11/24) TS^c^	12.5 % (1/8) WM^a^ 0 % CS^b^ 0 % TS^b^	0 % (0/6) WM 0 % (0/6) CS (0 %) (0/6) TS
**Experiment 5** **(Battery cages)**	WM TS	PHL	2/6	61.1 % (22/36) WM^a^ 16.7 % (6/36) TS^b^	41.6 % (5/12) WM 4.2 % (2/12) TS	2 % (1/36) WM^a^ 0 % (0/6) TS^b^

^1^*CS: low nutrient density diet, corn-soy based*.

^2^*WM: low nutrient density diet, wheat middlings based*.

^3^*TS: diet surpassing the nutritional requirements of young turkeys, turkey starter diet*.

^a−c^*Values within columns in each Experiment with different superscripts differ significantly (P < 0.05)*.

In pilot experiment 1, no horizontal transmission was observed (Table 2). In pilot experiment 2, the horizontal transmission was achieved, with 30.4% (7 out of 23) of the contacts presenting lesions in the ceca and/or liver, with only one mortality. Mortality of the seeder turkeys started on day 39 (21 d.p.i.), reaching 40% on day 44 (26 d.p.i.) when all seeders were humanely euthanized (Table 2). The trial was terminated on day 52 (34 d.p.i.).

**Figure 1:**
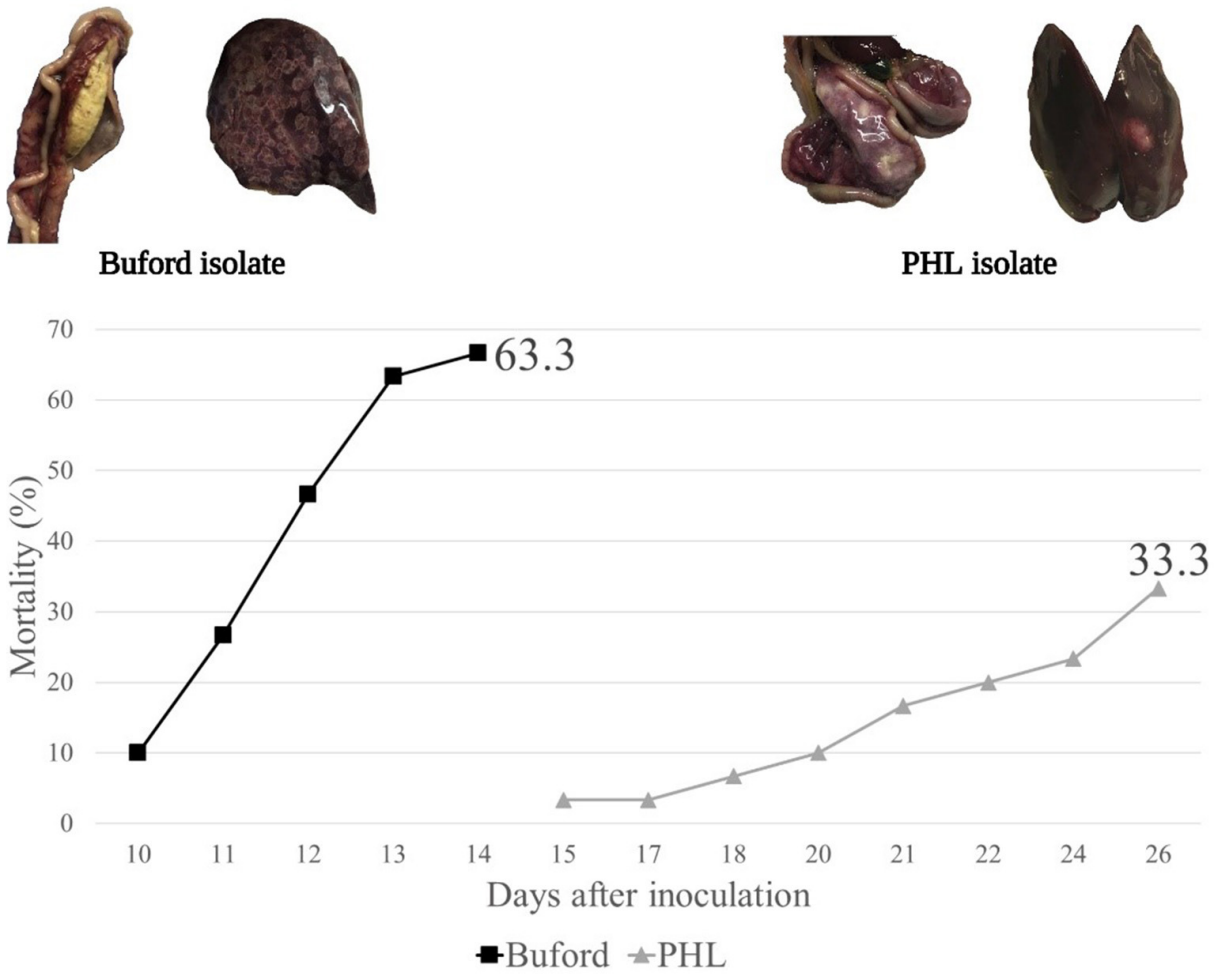
Mortality of turkeys directly inoculated (intra-cloacal) with two different isolates of *Histomonas meleagridis* (Buford or PHL) in experiment 3. Created with BioRender.com.

In Experiment 3, no horizontal transmission was observed in turkeys fed with WM and challenged with either PHL or Buford strain of *H. meleagridis*. Interestingly, turkeys fed with CS and challenged with the PHL isolate showed 57% of horizontal transmission in the contacts (Table 2). Moreover, this group had 23.3% (7 out of 30) mortality in the contact turkeys (Table 2). The positive control groups PC-Buford-WM and PC-PHL-WM had mortality rates of 63.3 and 33.3%, respectively, with mortality of turkeys on the PC-Buford-WM group starting at 10 d.p.i. and having a sharp increase until 14 d.p.i., while mortality on the group PC-PHL-WM started 15 d.p.i., prolonging until 26 d.p.i. (Figure 1). Mortality in the seeders of the HT-Buford-WM group began at 11 d.p.i., 15 d.p.i. in the PC-PHL-WM group, 16 and 21 d.p.i. in the groups HT-PHL-WM and HT-PHL-CS, respectively. Mortality of the seeder turkeys in the HT groups was 42.9% in the HT-PHL-CS and HT-PHL-WM groups, 57.1% in HT-Buford-CS, and 64.3% in HT-Buford-WM (Table 2, *P* > 0.05). From day 7 to 21, the groups NC-WM and NC-CS had a similar BWG, differing from the group PC-Buford-WM and PC-PHL-WM (Table 3). From day 7 to 38, the group NC-WM had a higher BWG than the group NC-CS (Table 3).

**Table 3:** Evaluation of body weight gain (BWG) or feed intake (FI) of turkeys inoculated with two different isolates of *Histomonas meleagridis* (Buford or PHL) and fed different experimental diets in validation experiments 3, 4 and 5.

**Experiment 3**	**BWG (d7-21)**	**BWG (d7-38)**
**NC** ^**1−**^ **WM** ^**2**^	233.1 ± 2.65^a^	870.9 ± 16.31^a^
**NC-CS** ^**3**^	233.6 ± 5.89^a^	610 ± 11.97^b^
**PC** ^**4**^ **-Buford-WM**	176.7 ± 11.86^c^	ND
**PC-PHL-WM**	210.1 ± 6.95^b^	ND
**Experiment 4**	**BWG (d7-30)**	
**NC-TS**	701.0 ± 10.44^b^	ND
**NC-WM**	834.0 ± 20.86^a^	ND
**NC-CS**	524.7 ± 16.03^c^	ND
**HT-TS**	586.0 ± 22.63^c^	ND
**HT-WM**	599.7 ± 29.02^c^	ND
**HT-CS**	425.0 ± 15.91^d^	ND
**Experiment 5**	**BWG, g (d9-27)**	**FI, g (d9-27)**
**NC-TS**	497.7 ± 7.48^a^	751.2 ± 21.96^a^
**NC-WM**	307.5 ± 8.07^c^	608.3 ± 10.61^b^
**HT-TS**	442.5 ± 20.09^b^	761.3 ± 40.51^a^
**HT-WM**	215.0 ± 22.22^d^	561.7 ± 24.26^b^

^1^*NC: non-challenge control*.

^2^*WM: low nutrient density diet, wheat middlings based*.

^3^*CS: low nutrient density diet, corn-soy based*.

^4^*PC: positive control (all turkeys directly inoculated on day 10 with the Buford or PHL isolates, 10^5^ histomonads/turkey, intracloacally)*.

*ND, not determined; d, day*.

*Data express as Mean ± SE. ^a−c^Values within columns with different superscripts differ significantly (P < 0.05)*.

In Experiment 4, the horizontal transmission was observed in all groups fed with different diet compositions (HT-TS, HT-WM, HT-CS). However, the group fed the turkey starter diet (HT-TS) had a lower percentage of contacts (*P* > 0.05) with lesions compared to both low-nutrient density diets (Table 2). In one of the four replicate cages of the group HT-TS, no contacts had cecal nor hepatic lesions, while both seeders presented cecal lesions. No hepatic lesions were observed in both seeders and contacts from all groups. Only one seeder from the HT-WM group died (Table 2). Mortalities were not observed in the other groups. From day 7 to 30, the group NC-WM had the highest BWG, followed by the NC-TS, HT-WM, HT-TS, NC-CS, and HT-CS (Table 3).

In Experiment 5, the horizontal transmission was observed in both groups (HT-TS and HT-WM). Agreeing with the previous experiment, a lower transmission level was observed in the group fed the turkey starter diet (HT-TS; Table 2). Four of six replicate cages of the HT-TS group had no contacts with lesions in the ceca or liver, although both seeders developed severe lesions, except one in one cage where only one seeder had lesions. The contacts of two cages of the HT-WM did not develop lesions in the ceca and liver. Two seeders died in the HT-TS group and five seeders and one contact died in the HT-WM group. The BWG from day 7 to 27, the group NC-TS had the highest BWG, followed by HT-TS, NC-WM, and HT-WM. Feed intake followed a similar pattern, being higher in the NC-TS and HT-TS groups, followed by NC-WM, and HT-WM (Table 3).

## Discussion

In the present study, variation was observed between experiments conducted under similar conditions. In Experiment 3, we could not achieve horizontal transmission using the same isolate (PHL) and diet (low-nutrient density wheat middlings diet) used in the pilot experiment 2. In addition to that, variability was observed within treatments in experiments 4 and 5 conducted in battery cages, with the horizontal transmission not being observed in some cages, although having the same conditions, agreeing with the variability of horizontal transmission observed in other studies (11).

Armstrong and McDougald (11) investigated the rate of transmission of histomonosis between turkeys exposed to directly inoculated turkeys (referred to as seeders) or to contaminated cages, where directly inoculated turkeys were previously present. The authors also compared the transmission rate between turkeys raised on bare-wire cage floors, paper-covered cage floors, or on floor pens with pine shavings. Differences were not detected in the rate of transmission between groups of turkeys placed on cages covered with paper or on floor pens with pine shavings. A higher rate of transmission was observed with turkeys that had directly contacted seeders, while turkeys exposed to contaminated cages had a lower infection rate. Interestingly, in one of three experiments conducted in similar conditions, having two seeder birds and six contact turkeys in battery cages with the floor covered, contact turkeys were not infected (11). Still, the seeders developed severe lesions of histomonosis, similarly to what was observed in some cages of experiments 4 and 5 of the present study. The variation in infection rate was not explained by the authors. McDougald and Fuller (12) compared the horizontal transmission rate of histomonosis in turkeys on battery cages. Each cage had a total of eight poults, with two, three, or four of them being directly inoculated with *H. meleagridis*, and each treatment had three replicates. The horizontal transmission was achieved in all groups, with 72.2, 80.0, and 75.0% of the contacts positive for histomonosis in the groups with two, three, and four seeder turkeys, respectively. The authors also investigated the impact of the length of exposure on contamination. Poults were exposed to seeders birds (two seeders and six contacts) for one, two, three, or four days. Each treatment had two replicates. Contact turkeys presented lesions at a rate of 16.7, 100, 87.5, and 100% when exposed to seeder turkeys for 1, 2, 3, or 4 days, respectively. The authors presented the results as the average of the replicates, not stating if variation was observed between replicates. Nevertheless, no information about the diets was provided (12). In a study by Landman and colleagues (13), ten turkeys were directly inoculated with *H. meleagridis* and 30 turkeys were exposed to them for 14 days. Seeders and contact turkeys were 14 days old. All contact turkeys were positive for histomonosis (mortality or lesions) at the end of the experiment. Only one study was reported without replication. Turkeys were fed a standard turkey feed containing 2292 cal/gram and 25.8% crude protein, but once again, no dietary ingredients were reported (13).

Variability in the incidence and outcome of histomonosis in outbreaks in turkey farms is also commonly reported, with only one house within a farm with multiple houses being affected; only one sex in mixed flocks, or only one section within a house (14, 15). Sulejmanović and colleagues (15) reported three outbreaks of histomonosis in turkey houses where toms and hens were raised together but in different compartments separated by a wire mesh. In the three outbreaks, only toms were severely affected by histomonosis. At the same time, female turkeys were infected, detected by the presence of antibodies evaluated by ELISA but did not manifest clinical signs. The presence of histomonads was also confirmed in high numbers in dust samples by PCR. The authors hypothesized that the gut microbiota and a variation in the immune response between males and females could be responsible for the difference in the disease outcome (15). Unfortunately, the stocking density was not reported in the two compartments of the house, nor the composition of the diets nor if the diets were different among males and females (15).

It is still not clear if histomonosis can be transmitted by contacting fresh feces in the litter or only by direct cloacal contact between turkeys and which factors would affect the survival of histomonads in the litter. Lotfi and colleagues (16) showed that histomonads could survive 9 h in turkey feces and non-chlorinated water, raising the possibility that the protozoa can be transmitted by contact with the litter and other environmental sources. In the present study, both pilot studies (Experiments 1 and 2) and Experiment 3 were conducted in floor pens with wood shavings as bedding material. Although it was not quantified, in pilot Experiment 2, litter moisture was apparently higher than in Experiment 3 in the floor pen of the group HT-WM. Moreover, in Experiment 3, a difference was noticed in the quality of the feces between groups fed the LOW diets, the corn-soy based or the wheat-middlings based, with turkeys from the HT-PHL-CS group presenting watery feces. Interestingly, that was the only group that experienced horizontal transmission. It is noteworthy that although the same diet formulation for the wheat middling diet was used in experiments 2, 3, 4, and 5, the nutritional composition of the diets varied and that in experiment 5, the WM diet had the addition of 3% of celite as a filler (Supplementary Table 1a). Another hypothesis, although not evaluated, is that the diets used in the different experiments had ingredients with different levels of mycotoxins. It is known that mycotoxins interact with the intestinal microbiota, possibly leading to dysbiosis (17), which could potentially favor the development of histomonosis. Only one study investigated horizontal transmission of histomonosis in turkeys fed a diet containing ingredients contaminated with aflatoxins (18). The influence of other mycotoxins in histomonosis is unknown and requires further investigation.

*Histomonas meleagridis* has a particular relationship with bacteria (19–21). Some studies suggest that bacteria can serve as a food source or provide specific compounds necessary for *H. meleagridis* survival (22–24). In other intestinal protozoa affecting human beings, such as *Entamoeba histolytica* and *Trichomonas vaginalis*, the bacteria can affect the virulence and adhesion of the protozoa (25, 26). In the case of *H. meleagridis*, the protozoa are probably influenced by the intestinal microbiota and the litter microbiota (22–24, 27). There is a direct correlation between gut and litter microbiota, and both are the reflection of the interaction between feed, ventilation, air quality, water quality, gender, among others (22–24, 27). One hypothesis is that depending on the bacteria and consequently by-products of bacterial fermentation present in the litter and ceca, the ability of *H. meleagridis* to adhere and invade host cells can be impaired. *Histomonas meleagridis* is a pleomorphic microorganism, assuming a rounded, flagellated form on the cecal lumen, transitioning to amoeboid during the invasion of tissues (28, 29). Under challenging conditions, the shape and behavior of histomonads can change to a cyst-like stage (28–30). The role of this cyst-like stage in the infectivity and transmission of histomonosis is unknown. One hypothesis is that the ability to invade tissue, causing infection, is reduced in these stages. Callait-Cardinal and colleagues (14) evaluated factors impacting the incidence and severity of histomonosis in free-range turkey flocks in France and the authors observed an interaction between hygiene and litter quality to the presence and severity of histomonosis. The authors hypothesized that higher levels of moisture in the building, caused by diarrhea, poor hygiene, and wet litter, could increase the contact between turkeys and their excreta.

To remove the variability of the litter, experiments 4 and 5 were conducted in battery cages with heavy paper covering the floor, allowing contact of turkeys with excreta. Nevertheless, variability was observed within treatments, which are puzzling findings. It could be hypothesized that the fecal moisture was different within treatments, impacting the survival or activation of histomonads; however, the position of each replicate cage in the room was randomized, therefore, a ventilation effect is not probable. Water consumption was not measured, but comparing the feed consumption of experiment 5, there was a low variation within treatments, suggesting that it is unlikely the possibility of some cages having a lower water consumption.

Interestingly, the BWG of turkeys eating the LOW, WM diet was higher than the BWG of turkeys eating the turkey starter in the experiment. The same was not observed in Experiment 5. In experiment 5, poor poult quality was observed, with 4.2% seven-day mortality. All groups were fed the turkey starter diet during the first seven days and poor poult quality reflected on the overall performance, as can be observed comparing the BWG of experiments and 5. The effect on BWG does not seem to be associated with transmission of histomonosis since, in experiment 4, turkeys in the HT-TS group had no difference in BWG compared to the HT-WM group; however, the HT-TS had a lower number of contacts presenting lesions. Poor poult quality has been linked with the severity of histomonosis (31).

Regarding the two isolates of *H. meleagridis* used in the present study, we were not able to achieve horizontal transmission with the Buford isolate, only with the PHL isolate. The Buford isolate was recovered from layer pullets around 20 years ago and supplied to us by Dr. Lorraine Fuller, University of GA, Athens. It is possible that although the isolate is still able to cause clinical disease and that initially, it led to horizontal transmission in experiments conducted by other research groups (personal communication); the isolate lost its ability to infect other turkeys by direct transmission during sequential *in vitro* passages during the years. The PHL isolate is contemporary, and it was isolated from turkeys. The Buford isolate is more virulent than the PHL isolate, causing the formation of cecal cores and inflammation of the ceca, together with severe hepatic lesions, and having a shorter incubation period compared with the PHL isolate. The PHL isolate causes severe typhlitis, much more severe than the one caused by the Buford isolate, sometimes leading to perforation of the cecal wall and peritonitis. Although the present study did not evaluate the genetic variation between the isolates Buford and PHL, based on the mortality rate, clinical signs, and lesions, it can be assumed that the isolates belong to different genetic clusters. In outbreaks in commercial flocks, usually, a combination of isolates of *H. meleagridis* can be involved in an infection, potentially explaining the variability in disease manifestation (32).

To conclude, we were able to reproduce horizontal transmission of histomonosis in four out of five experiments, more consistently on battery cages with the floors covered with paper and with diets with low-nutrient density. Transmission of histomonosis is multifactorial and not fully understood. Further studies are needed to investigate the role of litter moisture, diets, and morphological stages of *H. meleagridis* on the transmissibility of histomonosis.

## Data Availability Statement

The raw data supporting the conclusions of this article will be made available by the authors, without undue reservation.

## Ethics Statement

The animal study was reviewed and approved by University of Arkansas Agricultural Institutional Animal Care and Use Committee (Animal Use Protocol #19118).

## Author Contributions

TB and BH designed the experiments with inputs from EM, CV, JL, GT-I, and SR. TB conducted the experiments with assistance from RC, CV, JL, and GT-I. EM and SR assisted with the formulation of the experimental diets. TB, CV, RC, JL, and GT-I conducted termination of experiments and evaluated lesions. TB conducted the data analysis and wrote the manuscript with the support of all authors. All authors contributed to the article and approved the submitted version.

## Funding

The research was supported in part by funds provided by USDA-NIFA Sustainable Agriculture Systems, Grant No. 2019-69012-29905. Title of Project: Empowering U.S. Broiler Production for Transformation and Sustainability USDA-NIFA (Sustainable Agriculture Systems): No. 2019-69012-29905.

## Conflict of Interest

EM is employed by Cargill Turkeys LLC. The remaining authors declare that the research was conducted in the absence of any commercial or financial relationships that could be construed as a potential conflict of interest.

## Publisher's Note

All claims expressed in this article are solely those of the authors and do not necessarily represent those of their affiliated organizations, or those of the publisher, the editors and the reviewers. Any product that may be evaluated in this article, or claim that may be made by its manufacturer, is not guaranteed or endorsed by the publisher.
