# Supplementary material for: Feed Composition and Isolate of Histomonas meleagridis Alter Horizontal Transmission of Histomonosis in Turkeys. Proof of Concept
Source: Front Vet Sci. 2022 Jun 28;9:937102. doi: 10.3389/fvets.2022.937102 (PMC9277661; doi:10.3389/fvets.2022.937102)
Supplement: Supplementary file 1 [file Table_1.docx]

Supplementary Material

Table 1a. Analysis (*as-is* basis) of the three different batches of wheat middling based low-nutrient diet used in experiments 2, 3, 4, and 5.

| **Items** | **Experiment 2^§^** | **Experiment 3^£^** | **Experiment 4^¥^** | **Experiment 5^€^** |
| --- | --- | --- | --- | --- |
| **HT^1^ achieved** | Yes | No | Yes | Yes |
| **DM, %** | 89.3 | 89.6 | 90.2 | 90.8 |
| **Crude protein, %** | 14 | 17.4 | 19.7 | 16.4 |
| **Gross energy, kcal/kg** | 3,858 | 3,934 | 3,960 | 3,805 |
| **Fiber NDF, %** | 17.4 | 12.3 | 12.3 | 15.9 |
| **Fiber ADF, %** | 6.36 | 4.05 | 3.7 | 5.83 |
| **Al, mg/kg** | 109 | 75 | 49.1 | 169 |
| **Ca, mg/kg** | 10,743 | 9,941 | 9,014 | 14,133 |
| **Cu, mg/kg** | 123 | 20.4 | 31.7 | 52.4 |
| **Fe, mg/kg** | 247 | 125 | 122 | 517 |
| **K, mg/kg** | 4,666 | 5,291 | 5,974 | 5,568 |
| **Mg, mg/kg** | 1,672 | 1,760 | 1,767 | 1,868 |
| **Mn, mg/kg** | 131 | 164 | 149 | 181 |
| **Na, mg/kg** | 1003 | 887 | 1051 | 1,242 |
| **P, mg/kg** | 7,857 | 7,994 | 7,619 | 9,715 |
| **S, mg/kg** | 1,810 | 2,237 | 2,504 | 2,090 |
| **Zn, mg/kg** | 532 | 172 | 136 | 376 |

| HT: horizontal transmission of histomonosis.  ^§^Experiment 2: inoculation of the PHL isolate on day 18 in 10 seeders (34 contacts). Poults fed the TS diet for the first 14 days and, on day 15, the WM diet was introduced until the end of the experiment (day 52).  ^£^Experiment 3: inoculation of Buford or PHL on day 10 in 14 seeders (31 contacts) in a floor pen. All groups fed the TS diet the first seven days, then the WM diet from day 7 to 38 (groups 2, 3, 4, 5, 7), or the CS diet, divided into two phases (CS1 and CS2), from d7-21 (CS1) and d21-38 (CS2) (groups 1, 6, and 8).  ^¥^Experiment 4: inoculation of PHL isolate on day 9 in 2 seeders/cage (6 contacts/cage, 4 cages). All groups fed the TS diet for the first seven days, then the WM diet was introduced on day 7 until termination (d30) (groups 2 and 5), or the CS diet was divided into two phases (CS1 and CS2), the CS1 from day 7 to day 21 and CS2 from day 21 to termination (d30) (groups 3 and 6), or the TS the whole period (groups 1 and 4).  ^€^Experiment 5: inoculation of PHL isolate on day 9 in 2 seeders/cage (6 contacts/cage, 6 cages). All groups fed the TS diet for the first seven days, then the WM diet was introduced on day 7 until termination (d29) (groups 2 and 4), or the TS diet for the whole period (groups 1 and 3). |
| --- |
